# Acute Obstructive Hydrocephalus Due to Cysticercosis During Pregnancy

**DOI:** 10.1155/S1064744994000086

**Published:** 1994

**Authors:** Ronald M. Ramus, Mark Girson, Diane M. Twickler, George D. Wendel

**Affiliations:** 1 Department of Obstetrics and Gynecology University of Texas Southwestern Medical Center 5323 Harry Hines Boulevard Dallas TX 75235-9032 USA; 2 Department of Radiology University of Texas Southwestern Medical Center Dallas TX USA

## Abstract

*Background:* Cysticercosis, due to the parasite *Taenia solium*, can involve any organ. When central nervous system infection occurs, signs and symptoms depend on the location of the cerebral lesions. Most patients develop seizures, focal symptoms, or headaches with nausea and vomiting.

*Case:* A case of extraparenchymal (intraventricular) cysticercosis was diagnosed in a patient at term who presented with acute alteration in mental status. Ventriculostomy was performed because of acute obstructive hydrocephalus. Labor ensued and was augmented with oxytocin. Intrapartum management included magnesium sulfate seizure prophylaxis and corticosteroids. Intracranial pressures ranged between 4 and 12 cm H_2_O peripartum with approximately 300 mL of cerebrospinal fluid drained over the first 24 hours. Postpartum management included craniotomy with resection of a larval cyst and oral praziquantel therapy.

*Conclusion:* This case describes an uncommon presentation of neurocysticercosis that should be considered in gravidas with acute mental status changes.


Infectious Diseases in Obstetrics and Gynecology 1:198–201 (1994)

© 1994 Wiley-Liss, Inc.

### Acute Obstructive Hydrocephalus Due to Cysticercosis During Pregnancy

**Ronald M. Ramus, Mark Girson, Diane M. Twickler, and George D. Wendel, Jr.**

*Departments of Obstetrics and Gynecology (R.M.R., G.D.W.) and Radiology (M.G., D.M.T.), University of Texas Southwestern Medical Center, Dallas, TX*

Abstract

***Background:*** **Cysticercosis, due to the parasite *Taenia solium*, can involve any organ. When central nervous system infection occurs, signs and symptoms depend on the location of the cerebral lesions. Most patients develop seizures, focal symptoms, or headaches with nausea and vomiting.**

***Case:*** **A case of extraparenchymal (intraventricular) cysticercosis was diagnosed in a patient at term who presented with acute alteration in mental status. Ventriculostomy was performed because of acute obstructive hydrocephalus. Labor ensued and was augmented with oxytocin. Intrapartum management included magnesium sulfate seizure prophylaxis and corticosteroids. Intracranial pressures ranged between 4 and 12 cm $H_2O$ peripartum with approximately 300 mL of cerebrospinal fluid drained over the first 24 hours. Postpartum management included craniotomy with resection of a larval cyst and oral praziquantel therapy.**

***Conclusion:*** **This case describes an uncommon presentation of neurocysticercosis that should be considered in gravidas with acute mental status changes.** © 1994 Wiley-Liss, Inc.

Key words

Neurocysticercosis, organic brain syndrome, *Taenia solium*

Cysticercosis is a parasitic disease caused by *Taenia solium*, the pork tapeworm. Humans are the only known host of the adult cestode, which resides in the intestine. Infection is acquired by eating undercooked pork infested with cysticerci. In contrast, both humans and pigs can serve as intermediate hosts for the larval stage, which can develop in almost any tissue in the body, and is responsible for the clinical manifestations of this infection. The routes by which a human may become an intermediate host include contact with food or water contaminated with human feces (the most common source), autoinfection from anus to mouth by a person with the adult tapeworm, or reverse peristalsis and internal infection. Cysticercosis is rare, but is the most common parasitic disease affecting the central nervous system,[1] with a variety of clinical presentations. Most cases of neurocysticercosis have a relatively benign course and are due to cysts in the cerebral parenchyma. Extraparenchymal disease (intraventricular, subarachnoid, and spinal) is due to cysts in the cerebrospinal fluid and carries a poorer prognosis. This report describes a case of intraventricular cysticercosis that presented as acute obstructive hydrocephalus in a patient with a term gestation. Upon review of the literature, we found this case to be the second reported one of extraparenchymal neurocysticercosis associated with pregnancy.

### CASE REPORT

The patient is a 28-year-old Hispanic female, $G_3P_2$, who initially presented for prenatal care at 19 weeks

**Address correspondence/reprint requests to Dr. Ronald M. Ramus, Department of Obstetrics and Gynecology, University of Texas Southwestern Medical Center, 5323 Harry Hines Boulevard, Dallas, TX 75235-9032.**

Received September 28, 1993
Accepted December 17, 1994

gestation. Her past medical history was unremarkable. She had delivered term infants in 1978 in Mexico and in 1987 in Dallas, after moving to the United States from Mexico in 1984.

The patient's prenatal course was remarkable for acute right adnexal torsion at 25 weeks gestation necessitating exploratory laparotomy and right salpingo-oophorectomy. Her postoperative course was uncomplicated, and she was discharged on the fifth postoperative day.

At 38.5 weeks gestation, the patient was brought to the emergency room by her family because of acute mental status changes. Two hours previously, the patient began mumbling inappropriate responses to questions and became progressively less responsive. There were no seizures, but the patient complained of a severe headache several hours prior to her mental status changes. There was no history of nausea or vomiting.

On examination, the patient was obtunded and unresponsive to stimuli. Her oral temperature was 38.4°C, blood pressure 170/90 mmHg, pulse 100, and respirations 24/min. Her pupils were sluggishly reactive, horizontal nystagmus was present, and her left eye was outwardly deviated. Mild papilledema was present. No other focal neurologic findings were present. The patient could move all extremities, and reflexes were normal. Fetal heart tones were 150 beats/min. Her cervix was 1 cm dilated and 25% effaced. Cephalic presentation was noted. There were no subcutaneous or intramuscular nodules.

The patient's white blood cell count was 11,300/mm$^3$, hemoglobin, 10.7 g/dL; hematocrit, 32.0%; and platelet count, 247,000/mm$^3$. Electrolytes, liver function tests, urinalysis, and coagulation studies were all normal. Arterial blood gas on room air was pH, 7.47; $pCO_2$, 27 mmHg; $pO_2$, 132 mmHg; with an oxygen saturation, 99%. The chest x-ray was normal.

The patient was admitted to labor and delivery, and an emergency computed tomography (CT) scan of the head was obtained. It demonstrated hydrocephalus with dilatation of the lateral and third ventricles. A soft tissue density was present within the posterior portion of the third ventricle, representing the intraventricular cysticercus cyst (Fig. 1). Several parenchymal cysticercus cysts were present, manifest by low density areas with punctate high density scoleces.

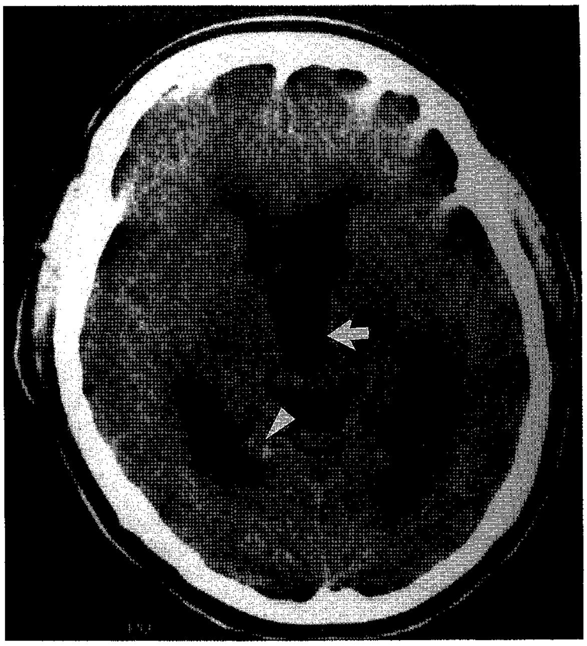

**Fig. 1. Axial CT scan on admission. There is hydrocephalus, manifested by dilation of the third and lateral ventricles, with periventricular calcifications (arrowhead). The intraventricular cysticercus cyst (arrow) is seen as a soft-tissue density within the dilated third ventricle.**

The patient was started on magnesium sulfate for seizure prophylaxis and intravenous dexamethasone. Magnesium sulfate was selected since it is the standard medication used for seizure prophylaxis in our labor and delivery unit.[2] At that time, protocols for the administration of alternative anticonvulsants had not yet been developed. Neurosurgical consultation was obtained, and a ventriculostomy shunt was placed in the right lateral ventricle to relieve the acute obstructive hydrocephalus. This was followed by a gradual return to normal neurologic function over 12–24 hours. Intracranial pressures were noted to vary between 4 and 12 cm $H_2O$ after ventriculostomy placement, with approximately 300 mL of cerebrospinal fluid drained over the next 24 hours. Ventricular cerebrospinal fluid studies including a cell count, protein, glucose, and cultures were performed post-ventriculostomy and were normal.

The patient's fever and hypertension present at admission spontaneously resolved. In addition, the patient began having regular contractions with cervical dilation to 2 cm. Oxytocin augmentation of labor resulted in the delivery of a 2,745-g male

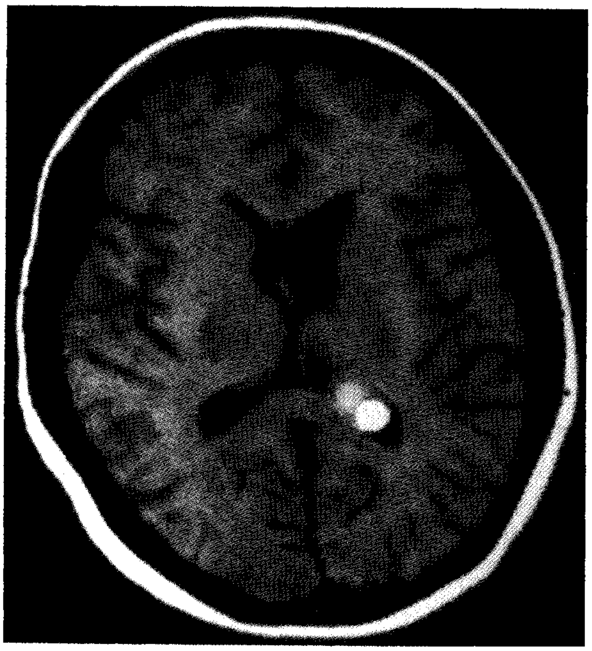

Fig. 2. MRI scan of the head following ventriculostomy. An axial T1 weighted image through the ventricles demonstrates the high signal intensity cysticercus cyst within the trigone region of the left lateral ventricle. There is disproportionate enlargement of the left lateral ventricle, but the right lateral and third ventricles are normal in size.

infant over an intact perineum, with Apgar scores of 8 and 9 at 1 and 5 minutes, respectively.

Magnetic resonance imaging (MRI) scan of the head, performed 36 hours following ventriculostomy, showed the right lateral ventricle and third ventricle to be of normal size. The left lateral ventricle remained enlarged, and the cyst, previously seen within the third ventricle, was now apparent posteriorly in the left lateral ventricle. This suggested migration of the intraventricular cysticercus cyst from the third ventricle into the lateral ventricle. The cyst manifested high signal intensity on both T1 and T2 weighted sequences, with respect to cerebrospinal fluid and brain parenchyma (Fig. 2). The parenchymal cysticercus lesions, seen previously on CT scan, were once again seen as cystic structures with the high signal intensity scoleces.

The ventriculostomy was removed 6 days after placement, and the next day craniotomy was performed with resection of an intact larval cyst measuring 2.0 × 1.5 × 1.5 cm. The patient's postoperative course was unremarkable. She was discharged on the fifth postoperative day on diphenylhydantoin and a 14-day course of praziquantel.

### DISCUSSION

There are a variety of clinical manifestations of central nervous system (CNS) cysticercosis. Seizures, focal neurologic deficits, decreased visual acuity, and altered mental status have all been described.[3] The particular symptoms in any patient depend primarily upon the anatomic location of the cysts. In addition, the degree of associated inflammation and edema can influence the clinical picture. The cysts usually remain viable for 3–5 years and then degenerate, which can induce a significant inflammatory response.

The diagnosis of cerebral cysticercosis is usually made by CT or MRI of the CNS.[4–6] Cerebrospinal fluid (CSF) studies can also help diagnose and classify CNS cysticercosis. Nonspecific findings, such as increased CSF protein, lymphocytosis, and eosinophilia can be indicators of the severity of an inflammatory response in the subarachnoid space.[3] In addition, an immunologic response in blood or CSF to cysticercal antigens, as demonstrated in an enzyme-linked immunoelectrotransfer blot (EITB), is diagnostic. The EITB assay has a sensitivity of 95% and a specificity of 100%, and has been mostly utilized in areas of endemic infection where high resolution imaging techniques are impossible and many asymptomatic individuals with infection are present.[7]

The only other reported case of extraparenchymal neurocysticercosis during pregnancy occurred in a Mexican visitor to Arizona in 1972.[8] The patient presented at 12 weeks gestation with a 3-day history of nausea, vomiting, and headache. She became unresponsive and tachypneic and then respirations suddenly ceased. Upon arrival at the hospital, she was cyanotic with a weak pulse, no respirations, and pupils that were fixed and dilated. Aggressive supportive care was initiated with gradual deterioration of the patient's status and subsequent demise 24 hours after admission. Autopsy revealed cysticercosis of the right lateral ventricle and obstructive hydrocephalus secondary to cysticerci.

The optimal treatment of cysticercosis is prevention. Personal hygiene and sanitary health measures are critical to avoid human fecal contamination. In addition, the larvae are destroyed by either freezing or thoroughly cooking pork. In patients with neurocysticercosis, treatment is primarily determined by the anatomic location of the disease. Algorithms of proposed treatment options have been devised.[9] Inactive disease is usually characterized by granulomas or calcifications on imaging of the CNS, and only supportive measures are indicated. This would include anticonvulsants for those with recurrent seizures. Surgical removal of inactive neurocysticercosis or antihelminthic therapy is not indicated. Parenchymal disease from viable cysts can be treated medically with praziquantel[10] or albendazole.[11] There is good evidence that medical therapy is efficacious, with success rates as good as surgical therapy in reducing the frequency of subsequent seizures in patients with parenchymal infection.[12] Therapy is more complicated for extraparenchymal neurocysticercosis. Subarachnoid cysticercosis can also be treated medically after shunting for any associated hydrocephalus is accomplished. However, these patients do not respond as well to medical therapy and frequently require surgical removal of focal lesions.[3] Intraventricular cysticercosis often presents with hydrocephalus, requiring a ventricular shunt. Primary therapy of cysticerci is surgical, as anti-cysticercal drugs are not effective.[13] However, this concept has been recently challenged by a report of a patient with intraventricular cysticercosis successfully treated with praziquantel.[14] Spinal cysticercosis can be treated medically, with surgical resection by laminectomy reserved for persistent disease or cysts in a single location.[3] Most patients require seizure prophylaxis with anticonvulsants, and steroids may be utilized if associated cerebral edema is present.

The possibility of neurocysticercosis is most commonly considered by the obstetrician in the differential diagnosis of peripartum seizures, particularly in women from Mexico and South America.[15] However, as this patient illustrates, cysticercosis may also present with only acute mental status changes due to acute obstructive hydrocephalus. Aggressive medical and surgical intervention resulted in maternal survival and return of normal neurologic function. The diagnosis of neurocysticercosis should be considered in gravidas with signs and symptoms of an acute organic brain dysfunction.

### REFERENCES

1. Sotelo J: Neurocysticercosis. In Kennedy PGE, Johnson RT (eds): Infections of the Nervous System. London: Butterworth, pp 145–152, 1987.
2. Pritchard JA, Cunningham FG, Pritchard SA: The Parkland Memorial Hospital protocol for treatment of eclampsia: Evaluation of 245 cases. Am J Obstet Gynecol 148:951–960, 1984.
3. Del Brutto OH, Sotelo J: Neurocysticercosis: An update. J Infect Dis 10:1075–1087, 1988.
4. Suss RA, Maravilla KR, Thompson J: MR imaging of intracranial cysticercosis: Comparison with CT and anatomapathologic features. AJNR 7:235–242, 1986.
5. Ramos OM, Stiebel-Chin G, Altman N, Duchowny M: Diagnosis of neurocysticercosis by magnetic resonance imaging. Pediatr Infect Dis 5:470–473, 1986.
6. Barkovich AJ, Citrin CM, Klara P, Wippold FJ, Kattah J: Magnetic resonance imaging of cysticercosis. West J Med 145:687–690, 1986.
7. Garcia HH, Martinez M, Gilman R, et al.: Diagnosis of cysticercosis in endemic regions. Lancet 338:549–551, 1991.
8. Bazley WS: Maternal mortality due to cysticercus cerebri: A case report. Obstet Gynecol 39:362–367, 1972.
9. Castero I: Tratado de Anatomia Patologica. Mexico City: Ed Atlante, pp 1485–1495, 1946.
10. Sotelo J, Escobedo F, Rodriquez-Carbajal J, Torres B, Rubio-Donnadieu F: Therapy of parenchymal brain cysticercosis with praziquantel. N Engl J Med 310:1001–1007, 1984.
11. Escobedo F, Penagos P, Rodriques J, Sotelo J: Albendazole therapy for neurocysticercosis. Arch Intern Med 147: 738–741, 1987.
12. Vazquez V, Sotelo J: The course of seizures after treatment for cerebral cysticercosis. N Engl J Med 327:696–701, 1992.
13. Sotelo J: Cysticercosis: In Johnson RT (ed): Current Therapy in Neurologic Disease. Vol 2. Philadelphia: BC Decker, pp 114–117, 1987.
14. Bandres JC, White AC, Samo T, Murphy EC, Harris RL: Extraparenchymal neurocysticercosis: Report of five cases and review of management. Clin Infect Dis 15:799–811, 1992.
15. Brown CEL, Purdy P, Cunningham FG: Head computed tomographic scans in women with eclampsia. Am J Obstet Gynecol 159:915–920, 1988.

## References

[PDF_00314] Bandres J. C., White A. C., Samo T., Murphy E. C., Harris R. L. (1992). Extraparenchymal neurocysticercosis: report of five cases and review of management.. Clin Infect Dis.

[PDF_00291] Barkovich A. J., Citrin C. M., Klara P., Wippold F. J., Kattah J. (1986). Magnetic resonance imaging of cysticercosis.. West J Med.

[PDF_00297] Bazley W. S. (1972). Maternal mortality due to cysticercus cerebri. A case report.. Obstet Gynecol.

[PDF_00318] Brown C. E., Purdy P., Cunningham F. G. (1988). Head computed tomographic scans in women with eclampsia.. Am J Obstet Gynecol.

[PDF_00283] Del Brutto O. H., Sotelo J. (1988). Neurocysticercosis: an update.. Rev Infect Dis.

[PDF_00305] Escobedo F., Penagos P., Rodriguez J., Sotelo J. (1987). Albendazole therapy for neurocysticercosis.. Arch Intern Med.

[PDF_00294] Garcia H. H., Martinez M., Gilman R., Herrera G., Tsang V. C., Pilcher J. B., Diaz F., Verastegui M., Gallo C., Porras M. (1991). Diagnosis of cysticercosis in endemic regions. The Cysticercosis Working Group in Peru.. Lancet.

[PDF_00288] Ramos O. M., Stiebel-Chin G., Altman N., Duchowny M. (1986). Diagnosis of neurocysticercosis by magnetic resonance imaging.. Pediatr Infect Dis.

[PDF_00301] Sotelo J., Escobedo F., Rodriguez-Carbajal J., Torres B., Rubio-Donnadieu F. (1984). Therapy of parenchymal brain cysticercosis with praziquantel.. N Engl J Med.

[PDF_00285] Suss R. A., Maravilla K. R., Thompson J. (1986). MR imaging of intracranial cysticercosis: comparison with CT and anatomopathologic features.. AJNR Am J Neuroradiol.

[PDF_00308] Vazquez V., Sotelo J. (1992). The course of seizures after treatment for cerebral cysticercosis.. N Engl J Med.

